# Editorial: The potential effects and mechanisms of Chinese traditional medicine on bone homeostasis and remodeling, volume II

**DOI:** 10.3389/fendo.2023.1158042

**Published:** 2023-03-28

**Authors:** Ling-Feng Zeng

**Affiliations:** Guangdong Provincial Hospital of Chinese Medicine, Guangzhou University of Chinese Medicine, Guangzhou, China

**Keywords:** traditional Chinese medicine, bone homeostasis, bone remodeling, mechanisms, potential effects

## Introduction

Traditional Chinese medicine (TCM) is an important complementary and alternative medicine that provides more possibilities for the treatment of various diseases. Osteoporosis (OP) is a common metabolic bone disease in the clinic and is characterized by bone microstructure destruction, a reduction in bone density, and an increase in bone fragility, and fracture is a common complication (1). There are more than 8.9 million secondary fractures due to OP every year worldwide, and the proportion of women and men over 50 years old who have experienced OP fracture is one-third and one-fifth, respectively (2). Bisphosphonates, oestrogens, denosumab, teriparatide, calcium, and other drugs are commonly used in the treatment of OP (3, 4), but low compliance and objective adverse reactions must be considered regarding their use. In China, TCM, especially Chinese herbal medicine, is widely used in the treatment of OP and is suggested to increase bone density (5). However, the theory of TCM is relatively complex and lacks advanced evidence-based support. Moreover, the mechanism and clinical efficacy of the treatment of OP using TCM are still controversial, especially in clinical application scenarios in different countries, or with patients of different nationalities. Therefore, taking advantage of the achievements and methods of modern science and research theories, it is worthwhile to further clarify the efficacy and potential mechanism of TCM in the treatment of OP, the goal of this Research Topic. This Research Topic includes six articles that preliminarily explained the potential mechanism of some commonly used Chinese herbal compounds, herbs, and components in treating OP, showing their high potential with respect to drug transformation.

## Chinese herbal compounds in the treatment of OP

Chinese herbal compounds are the main method of treating diseases with TCM. Chinese herbal compounds can be composed of two or more types of herbal medicines, animal medicines, or mineral medicines, but they mainly comprise natural plants. Chinese herbal compounds have many drug components and complex chemical components, and their pharmacological action is characterized by multiple targets and multiple pathways. Therefore, research on Chinese herb compounds has always been a major challenge for the development of TCM. There are three types of Chinese herbal compounds collected in this Research Topic, namely, *Erxian Decoction* (EXD), *Yougui Yin* (YGY), and Qing’e Pills (QEP). EXD is usually used to treat OP, but its mechanism is unclear. In recent years, researchers have paid more attention to the study of OP and OA comorbidity. Ma et al. revealed that EXD can treat osteoarthritis (OA) by affecting cystine, chenodeoxycholate, and D-turanose and their associated glycolysis/gluconeogenesis, pantothenate, and CoA biosynthesis metabolic pathways, which also revealed the potential mechanism by which EXD improves the internal environment of OP. YGY is a classic Chinese herbal compound used for the treatment of OP, and the study of its mechanism will help promote the clinical promotion of this compound. Zeng et al. confirmed through *in vivo* and *in vitro* experiments that N-butanol extract of modified YGY (MYGY-Nb) significantly attenuates osteoclast formation by regulating the RANKL-mediated NF-κB signalling pathway, which is considered to be the key link through which MYGY-Nb maintains bone homeostasis. In recent years, the role of factors related to angiogenesis-osteogenesis in the treatment of OP has also attracted the attention of researchers. Lu et al. explored the anti-OP mechanism of modified QEP (MQEP) from the perspective of angiogenesis. Their research results showed that MQEP can promote H-type angiogenesis by promoting the expression of hypoxia-inducible factor 1 alpha and vascular endothelial growth factor, which is the potential mechanism by which MQEP reduces bone loss. This research provides a new idea for exploring the mechanism of TCM in treating OP.

## Rhizoma Drynariae (Flavonoids) and OP

Flavonoids are considered to have great potential for drug conversion in the treatment of OP and have been developed and applied in China. This Research Topic includes two studies on the mechanism of Rhizoma Drynariae or total flavonoids of Rhizoma Drynariae (TFDR) in the treatment of OP. Zhang et al. hypothesized that the potential effective chemical components of TFDR in the treatment of glucocorticoid-induced OP were luteolin-7-glucuronide, naringenin, naringenin chalcone, and eriodityol. Their conclusion is that TFDR can improve glucocorticoid-induced bone loss and bone microstructure damage by targeting the core target PPARγ related to lipid metabolism, which also provides a new perspective for understanding the pharmacological mechanism of TFDR in treating OP. In addition, Su et al. studied the anti-OP effect of Rhizoma Drynariae from the perspective of transcriptome sequencing. Their research results suggest that the Npas2, Dbp, Rt1, Arntl, Grem2, H2bc9, LOC501233, Pla2g2c, Hpgd, Pde6c, and Dner genes, as well as the circadian rhythm, lipid metabolism, inflammatory signalling pathway, and immune pathways may be potential factors related to the anti-OP effect of Rhizoma Drynariae. Although Su et al. have invested much effort in their research, a more specific, rather than such a broad research perspective is worth further consideration.

## *Yushen Hezhi* and OP

*Yushen Hezhi* therapy (YSHZT) is a common treatment modality for OP or OA with TCM (6). Zhao et al. carried out an umbrella review, and their research concluded that the methodological and evidence qualities of the published systematic evaluations (and/or meta-analyses) of YSHZT in postmenopausal OP (PMOP) are poor; therefore, the effectiveness and safety of YSHZT in the treatment of PMOP still need to be further verified by more high-quality research. Generally, high-quality systematic evaluation needs to be based on high-quality original research. To further verify the clinical value of YSHZT in treating PMOP, future research needs to design and implement higher-quality intervention trials.

## Conclusion

The six studies collected in this Research Topic explain the clinical value and drug conversion potential of Chinese herbal compounds, single herbal medicines, compounds, and the treatment of OP based on the theory of YSHZT from a richer dimension, which will undoubtedly further enhance researchers’ confidence in exploring the treatment of OP with TCM. However, we also noticed that the content included in this Research Topic is mainly basic research or theoretical discussion, while clinical randomized controlled trials are relatively scarce. Therefore, the clinical efficacy of TCM in treating OP still needs more supporting evidence from large clinical randomized controlled trials, a direction that needs more attention in the future.

## Author contributions

The author confirms being the sole contributor of this work and has approved it for publication.
